# Desmoid tumor of trapezius muscle: A case report

**DOI:** 10.1016/j.amsu.2021.103127

**Published:** 2021-12-01

**Authors:** Abdelhakim Harouachi, Abdelbassir Ramdani, Ayoub Kharkhach, Nada Akouh, Tariq Bouhout, Amal Bennani, Badr Serji, Tijani EL. Harroudi

**Affiliations:** a Surgical Oncology Department, Regional Oncology Center, Mohammed VI University Hospital, Oujda, Morocco; b Mohammed First University Oujda, Faculty of Medicine and Pharmacy Oujda, Oujda, Morocco; c Department of Pathology, Mohammed VI University Hospital, Oujda, Morocco

**Keywords:** Desmoid tumors, Trapezius muscle, Loco-regional extension, Complete resection

## Abstract

**Introduction:**

Desmoid tumors are benign fibrous entities developed from connective tissue, and they result from muscle fascia or aponeurosis. Surgical excision is the main pillar of treatment.

**Case report:**

A 29-year-old female patient presented with a left postero-lateral cervical swelling that had been evolving for 11 months. Cervical MRI showed a mass at the expense of the trapezium muscle measuring 41 × 68 × 81 mm. A biopsy of the mass was performed, concluding a desmoid tumor. The patient underwent a large resection of the tumor involving the left trapezius muscle. An immunohistochemistry staining was performed, demonstrating cytoplasmic labeling with anti AML antibody, and nuclear labeling of tumor cells with anti-beta-catenin antibody. The case was analyzed by a multidisciplinary committee, and it was decided to follow the patient for surveillance.

**Discussion:**

The localization of desmoid tumors in the trapezius muscle is extremely rare. The main risk of these benign lesions is infiltration of surrounding tissue leading to morbidity and mortality. Recurrence is a main feature of these tumors, even if complete excision has been performed. Unresectable desmoid tumors require medical and non-surgical treatment such as hormone therapy (tamoxifen), and chemotherapy with vinblastine and methotrexate.

**Conclusion:**

Desmoid tumor of trapezius muscle is classified as benign without metastatic power, and the main risk is infiltration of surrounding tissue.

## Highlights

• Desmoid tumors are benign fibrous entities developed from connective tissue.
• Desmoid tumors comprise only 12–15% of cases in the head and neck, and can occur at any age.
• The localization of desmoid tumors in the trapezius muscle is extremely rare.
• The symptomatology of a desmoid trapezius muscle tumor remains aspecific.
• Complete resection with clear miscroscopic margins is the treatment of choice for resectable symptomatic desmoid tumors.

## 1 Introduction

Desmoid tumors are benign fibrous entities developed from connective tissue, and they result from muscle fascia or aponeurosis [1]. These lesions belong to the group of mesenchymal tissue neoplasms, and present less than 0.3% of tumors [2]. They comprise only 12–15% of cases in the head and neck, and can occur at any age [3].

This rare condition, also called aggressive fibromatosis, is without metastatic power. They present a high risk of recurrence, and a potential for infiltration and invasion of surrounding structures. Surgical excision is the main pillar of treatment [1,2].

In this article, we report the clinical case of desmoid tumor of trapezius muscle, and refer to recent review of literature to discuss the difficulties of surgical management. This case has been reported following the SCARE criteria [4].

## 2 Case report

A 29-year-old female patient, gravida 1, para 1, with no significant pathological history, had presented herself in consultation complaining of a left cervical swelling that had been evolving for 11 months in a context of general state conservation. The mass was increased in size rapidly after the end of the breastfeeding period (three months). The clinical examination at admission found normal vital signs, and Body mass index of 26.3 kg/m^2^. Family history was unremarkable for cancer. The patient was used a combined oral contraceptive for menstrual regulation.

The cervical examination showed a postero-lateral mass of the left neck measuring 6 cm in diameter, painless, of firm consistency, unilobed, soft, adherent to the superficial and deep plane. There was no palpable cervical lymphadenopathy or inflammatory signs of the adjacent skin.

**Fig. 1:**
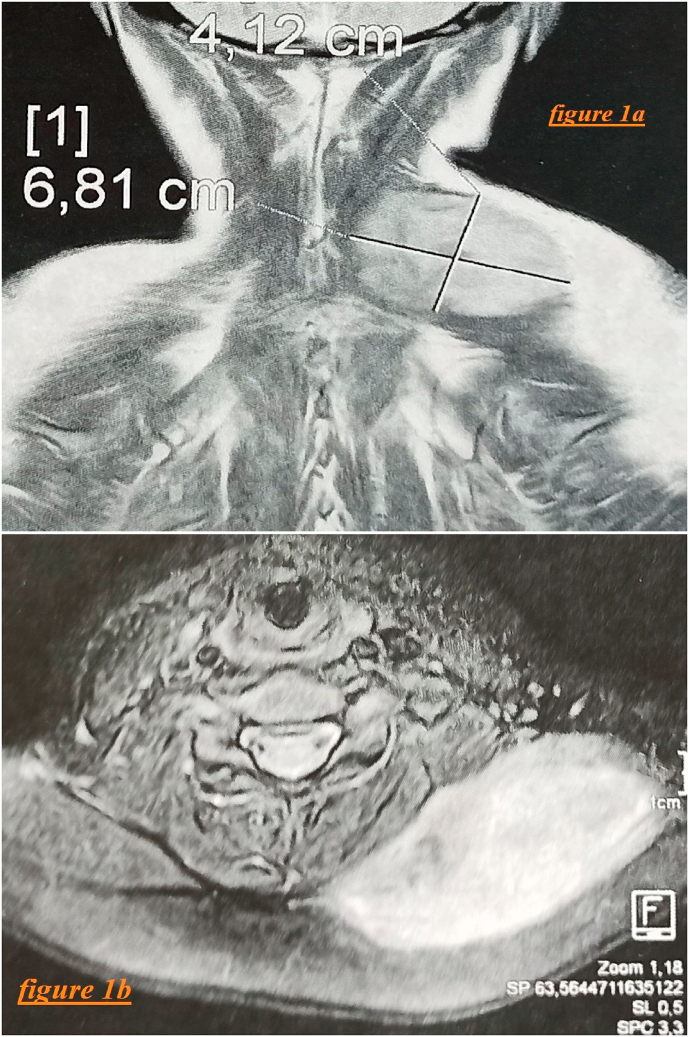
(1a and 1b): Cervical MRI showing mass of the left posterior cervical soft parts, at the expense of the trapezium muscle, measuring 41 × 68 × 81 mm.

A cervical magnetic resonance imaging (MRI) was performed, revealing a mass of the left posterior cervical soft parts, at the expense of the trapezium muscle, tissue, oval, of regular contours, well-defined, measuring 41 × 68 × 81 mm, enhanced after injection of Gadolinium, with multiple homolateral supraclavicular and lateral cervical lymph nodes (Fig. 1). The monotest, in the absence of an IRD tuberculin skin intradermoreaction, was negative.

A biopsy of the mass with Tru-cut® was performed, concluding a desmoid tumor. Abdominopelvic and thoracic computed tomography, indicated for staging, showed the absence of other progressive lesions elsewhere.

**Fig. 2:**
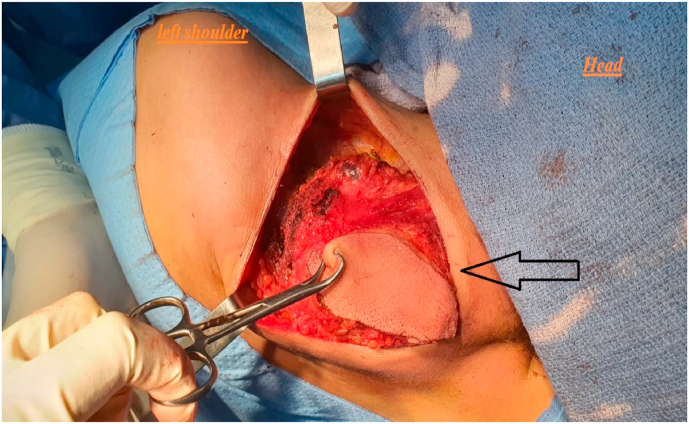
Intraoperative view of the desmoid tumor arising from trapezium muscle (arrow).

In view of these clinical, radiological and pathological findings, a large resection of the tumor was indicated. The patient was installed in the supine position. Access to the posterior neck area was via a direct surgical approach (Fusiform incision). The mass was found to originate from the trapezius muscle without local infiltration of surrounding structures. A sharp dissection over the mass of the tumor away from the muscle was not possible. Complete excision of the mass was accomplished involving the fascia and trapezius muscle (Fig. 2). A close suction drain was placed. The safety margins are macroscopically healthy with a resection at 1.5 cm macroscopic distance from the palpable area of the tumor. The post-operative outcomes were simple without any complications and the drain was removed on the second postoperative day. The patient was discharged from the hospital on postoperative day 5, and the functional result was considered satisfactory without any impairment noted.

**Fig. 3:**
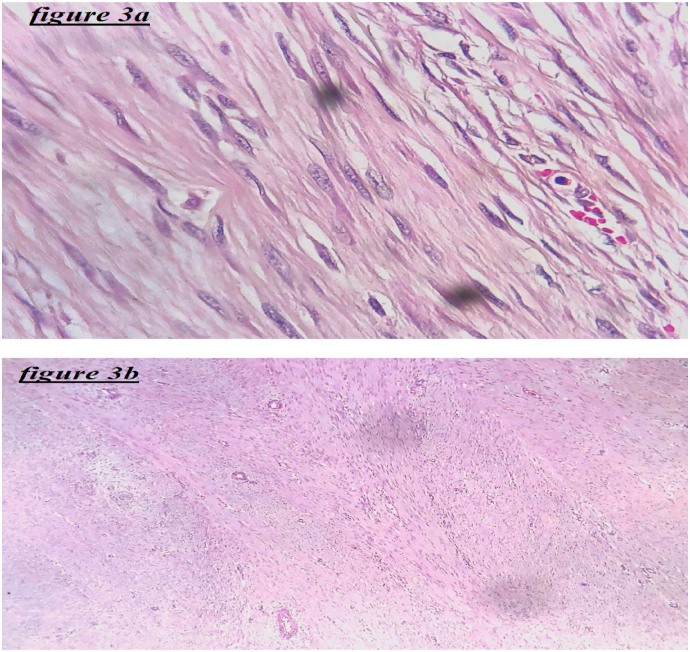
(3a and 3b): Microphotography showing fuso-cellular proliferation made up of long, crossed beams. Tumor cells have elongated nuclei and an eosinophilic cytoplasm with imprecise limits.

The pathology examination of the surgical specimen showed proliferation of spindle-shaped cells arranged in long fascicles in a collagenous stroma. This proliferation infiltrates the striated muscle, with negative excision margins. An immunohistochemistry staining was performed, demonstrating cytoplasmic labeling with anti AML antibody, and nuclear labeling of tumor cells with anti-beta-catenin antibody. This pathological and immunohistochemical aspect highlights the diagnosis of a desmoid tumor (Fig. 3).

The case was analyzed by a multidisciplinary committee, and it was decided to follow the patient without any adjuvant treatment given its long-term side-effects and the safety margins are healthy. After a three month, the clinical and radiological follow-up examinations were unremarkable. The outcome was favorable without local or distant tumor recurrence.

## 3 Discussion

Desmoid tumors are rare benign lesions with no metastatic potential resulting from the abnormal infiltrating proliferation of fibroblasts. Three types of these tumors can be distinguished: intra-abdominal form, the form of the abdominal wall, and extra-abdominal form. The localization of desmoid tumors in the trapezius muscle is extremely rare. The etiology of desmoid tumors is still poorly understood [5]. They are diagnosed mainly in adulthood with a high incidence in the age group from 24 to 40 years. A clear female predominance was noted [6].

Desmoid tumors can be sporadic as in our case or be part of familial adenomatous polyposis (FAP) or Gardner syndrome [5]. Lately, it is well established that estrogens are implicated in the genesis of these tumors, as well as the traumatic history. According to several studies, the incidence of desmoid tumors is increased after exposure to oral contraceptives, and during or after pregnancy [7]. The main risk of these benign lesions in the neck region is infiltration into critical structures such as vital neurovascular, leading to morbidity and mortality. Recurrence is the main feature of these tumors, even if complete excision has been performed [8].

The circumstances of the discovery of desmoid tumors are variable, and depend primarily on the size and location of the tumor. The clinical presentation of a desmoid tumor of the trapezius muscle remains aspecific. The desmoid tumor of the neck is presented in the majority of cases as a painless mass of hard consistency, gradually increasing in size [9]. The other main telltale symptoms are pain or a focal motor deficit when the nerve or muscle structures are affected, and dysphagia [15].

Computed tomography CT scan and MRI imaging confirm a site and a size of the lesion, suggest a diagnosis of desmoid tumor, and determine the locoregional extension and resectability of the tumor. The CT scan shows in most cases well-limited isodense lesions, homogeneously enhanced after injection of contrast product [10]. MRI is a valuable input in the exploration of desmoid tumors, allowing precise study of lesions and their relationship to adjacent structures such as the carotid or subclavian arteries. This tool shows a mass in isosignal compared to the muscles in T1-weighted sequence. In T2-weighted images, the signal is higher in the event of a myxoid component, in the event of large extracellular spaces, and in the event of high cellularity [11].

The complete resection with clear microscopic margins is the treatment of choice for resectable symptomatic desmoid tumors [12]. There is evidence that despite the fact that virtually the complete tumor excision is the optimal treatment choice, the real challenge for surgeon is the function-sparing resection and the infiltrative nature of the tumor in the head and neck region [15]. In our case, a complete resection of the mass removing the trapezius muscle was performed to avoid invasion of the vessels of the neck.

The radiotherapy, as primary treatment, is usually used for inoperable or inaccessible desmoid tumors. The Adjuvant radiotherapy is recommended to decrease the rate of spontaneous recurrence which is 33–75% of patients operated in 5 years, but its efficacy is controversial, given the fact of adverse side effects (secondary malignant transformation, neurological deficits …) [13,15].

The clinical and radiological follow-up should be indicated for asymptomatic desmoid tumors [12]. Unresectable desmoid tumors require medical and non-surgical treatment such as hormone therapy (tamoxifen), and chemotherapy with vinblastine and methotrexate. A combination of tamoxifen, anti-estrogenic, and meloxicam, a non-steroidal anti-inflammatory drug, aims to shrink the tumor (neo-adjuvant therapy) or stabilize the tumor (palliative) [14,15].

## 4 Conclusion

As in other locations, desmoid tumor of trapezius muscle is classified as benign, and the main risk is infiltration of surrounding tissue, leading to morbidity and mortality. This lesion is extremely rare that must be evoked in front of any isolated lateral cervical mass.

## Funding

The author(s) received no financial support for the research, authorship and/or publication of this article.

## Author contributions

**Dr Harouachi Abdelhakim:** Have written the article, have consulted the patient, prescribed all of the tests and prepared the patient for surgery and participated in the surgery.

**Dr Ramdani Abdelbassir:** have helped writing the article, data collection.

**Dr Kharkhach Ayoub:** have helped writing the article, data collection.

**Dr Akouh Nada:** Interpretation of pathological data.

**Pr Bouhout Tariq** (oncology surgery professor)**:** supervised the writing of manuscript.

**Pr Bennani Amal** (Pathology professor): confirm the histological diagnosis.

**Pr Serji Badr** (oncology surgery professor): Writing, review and editing of the manuscript, and had been the leader surgeon of the case.

**Pr El Harroudi Tijani** (oncology surgery professor): have supervised the writing of the paper.

## Ethical approval

No ethical approval necessary.

## Trial registry number

Our paper is a case report; no registration was done for it.

## Consent

Written informed consent was obtained from the patient for publication of this case report and accompanying images. A copy of the written consent is available for review by the Editor-in-Chief of this journal on request.

## Guarantor

Harouachi Abdelhakim.

## Provenance and peer review

Not commissioned, externally peer reviewed.

## Declaration of competing interest

The authors declared no potential conflicts of interests with respect to research, authorship and/or publication of the article.
